# Twenty-four months of bacterial colonialization and infection rates in patients with transcutaneous osseointegrated prosthetic systems after lower limb amputation—A prospective analysis

**DOI:** 10.3389/fmicb.2022.1002211

**Published:** 2022-10-31

**Authors:** Marcus Örgel, Horst-Heinrich Aschoff, Ludwig Sedlacek, Tilman Graulich, Christian Krettek, Sabine Roth, Alexander Ranker

**Affiliations:** ^1^Trauma Department, Hannover Medical School (MHH), Hannover, Germany; ^2^Institute for Medical Microbiology and Hospital Epidemiology, Hannover Medical School (MHH), Hannover, Germany; ^3^Department of Rehabilitation Medicine, Hannover Medical School (MHH), Hannover, Germany

**Keywords:** infection, bacterial colonization, transcutaneous osseointegrated prosthetic system (TOPS), amputation, osseointegration

## Abstract

**Background:**

Transcutaneous osseointegrated prosthesis systems (TOPS) are alternative rehabilitation methods to socket prosthetics, after limb amputation. TOPS compromise a two-step surgery: starting with the implantation of the stem which is then followed by the creation of the transcutaneous stoma through which the exoprosthesis can be connected. Immediately after surgery, this opening is permanently exposed to pathogens. This study aimed to investigate the dynamics of bacterial colonization of the stoma to analyze whether obligate bacterial colonization leads to a risk of periprosthetic infections after TOPS treatment.

**Methods:**

This prospective study analyzed data from 66 patients (aged 26–75 years) after TOPS treatment between 2017 and 2019. Microbiological swabs from the stoma were analyzed on the first postoperative day and 3, 6, 12, and 24 months after stoma creation. Infection rates, laboratory values (CRP, leukocyte count, hemoglobin), and body temperature were recorded at these points in time. Statistical analysis was performed using SPSS 28.

**Results:**

The results show the formation of a stable environment dominated by Gram-positive bacteria in the stoma of TOPS patients over 24 months. *Staphylococcus aureus, Staphylococcus spp*., and *Streptococcus spp*. were the most common species found. With regard to the cohort up to the 3 months follow-up, 7.9% (five patients) developed infections surrounding the TOPS procedure. In relation to the whole cohort with loss to follow-up of 80.3% at the 24 months follow-up the infection rates increased up to 38.3%.

**Conclusion:**

The soft tissue inside and around the transcutaneous stoma is colonialized by multiple taxa and changes over time. A stable Gram-positive dominated bacterial taxa could be a protective factor for ascending periprosthetic infections and could possibly explain the relatively low infection rate in this study as well as in literature.

## Introduction

The prevalence of amputation of extremities has increased considerably in the last years and is expected to rise even further in the upcoming years (Ziegler-Graham et al., 2008). Especially amputations of the lower limb, such as transfemoral amputations, result in a high deprivation of quality of life. The utilization of a prosthesis can restore participation in daily life and play the most crucial role in rehabilitation of patients with lower limb amputation (Frengopoulos et al., 2021; Knight et al., 2021). However, the standard socket suspension for attachment of the prosthesis has limitations (Cheifetz et al., 2007; Chislett et al., 2020). The socket prosthesis often leads to local irritation, and skin damage. Furthermore, weight-dependent changes of the soft tissue of the stump can lead to an insufficient attachment of the prothesis (Hoffmeister et al., 2017; Beck et al., 2019); but there are optional methods to attach the prosthetic leg to the tight stump. The treatment of amputations with transcutaneous osseointegrated prosthetic systems (TOPS) has been established throughout the last two decades in Germany, Sweden, the Netherlands, and Australia (Aschoff et al., 2009, 2010; Juhnke and Aschoff, 2015; Juhnke et al., 2015; Aschoff, 2017a,b; Pospiech et al., 2020). Compared to the most frequently used “socket suspension” method for prosthetic limb attachment, this method offers a variety of new options (Hagberg et al., 2005; Aschoff et al., 2009; Frossard et al., 2009; Van de Meent et al., 2013; Hoffmeister et al., 2017; Al Muderis et al., 2018). Furthermore, TOPS shows unique advantages, such as osseoperception, which leads to an improved sense of grounding with the prosthetic foot, and prosthetic limb control (Hagberg et al., 2008; Hagberg and Branemark, 2009; Örgel et al., 2021a). Another advantage is a higher physical functional performance with a better range of motion, a higher sitting comfort, and an unlimited “wearing time” of the prothesis (Hagberg et al., 2005).

Regarding the implantation, the surgical procedure is quite simple. The endo-fix stem is press-fitted into the bone in the first surgery, and after 4–6 weeks, the transcutaneous stoma is created in a second step (Örgel et al., 2021b). Currently, steps one and two are often performed in a single step together (Al Muderis et al., 2017; Reif et al., 2021). Despite all the advantages of this procedure, one major danger lies in the transcutaneous stoma which leads to the absence of a physiological skin seal which remains lifelong. This fact constantly exposes the implanted transcutaneous device to pathogens from the external environment.

Due to this circumstance, one may primarily assume a high-risk factor for infections. Interestingly the literature shows only low to moderate infection rates, which in most cases can be treated with antibiotic therapy (Tsikandylakis et al., 2014; Al Muderis et al., 2016; Hebert et al., 2017; Kunutsor et al., 2018; Atallah et al., 2020; Reetz et al., 2020; Wang et al., 2021). Infection rates differ between surgical centers, surgical techniques, and types of implants (Hagberg and Branemark, 2009; Aschoff et al., 2010; Tillander et al., 2010; Tsikandylakis et al., 2014; Al Muderis et al., 2016; Ranker et al., 2020). Besides tissue infections, rare cases of osteomyelitis, and bone necrosis were also observed (Tillander et al., 2010, 2017; Tsikandylakis et al., 2014).

Along with the low infection rates with external fixators, it remains unclear how the normal skin flora develops with TOPS. In a longitudinal cohort study, Beck et al. observed 10 patients with TOPS with skin and stomal swabs over a 1-year period (Beck et al., 2019). They showed that the microbiota on the stomal site develops over time and forms a stable skin flora with colonialization dominated by *Streptococcus spp., Corynebacterium spp*., and/or *Staphylococcus spp*.

As mentioned in the study from Örgel et al. (2022), this prospective study investigates the dynamics of bacterial colonization on the stomal site and elucidates whether treatment with TOPS leads to a large number (>10%) of periprosthetic and stomal infections. Laboratory values during the course were also monitored.

This leads to the following hypotheses:

- Null hypothesis: Treatment with a transcutaneous osseointegrated prosthetic system does not lead to a large number (>10%) of periprosthetic and stomal infections.
- Alternative hypothesis: Treatment with a transcutaneous osseointegrated prosthetic system leads to a large number (>10%) of periprosthetic and stomal infections.

## Methods

### Study design and participants

This prospective study followed the “Strengthening the Reporting of Observational Studies in Epidemiology (STROBE)” reporting guideline. Informed consent was obtained from each patient at all times. This paper reports the results of a data analysis within one cohort of patients treated with the Endo-Exo-Prosthesis (ESKA Orthopaedic Handels GmbH^®^, Osterweide 2c, 23562 Lübeck, Germany), which belongs to TOPS after transfemoral amputation. Overall, *n* = 66 patients were enrolled. All patients were treated surgically with TOPS between 2017 and 2019 in one center. The two-step procedure was performed in each case (Aschoff et al., 2010, 2011). Each patient was screened for oxacillin/methicillin-resistant *Staphylococcus aureus* colonization prior to surgical procedure. Idem to Beck et al., all patients received a single intravenous dose of cephazolin (2 g) for each surgery (step one and step two). Each dose was given within 30 min before incision (Beck et al., 2019). In cases of known penicillin allergy, clindamycin (600 mg) was used instead. Before each surgery, the skin was disinfected three times using Braunoderm (Braun Medical AG, Seesatz 17, 6204 Switzerland). The microbiological swabs, and the blood samples for monitoring the laboratory values were taken during the inpatient stay in the context of the operations, and the follow-up assessment in our outpatient clinic. The blood samples were analyzed for standardized laboratory values (c-reactive protein, leucocytes, hemoglobin, body temperature) on the first, third, and fifth, postoperative day, and 3, 6, 12, and 24 months after surgery. The microbiological swabs were collected during the second surgery (intraoperative), on the first day after the second surgery, and 3, 6, 12, and 24 months after surgery.

### Swab material and technique

Every patient was screened *via* stomal *microbial* sampling with a swab (Amies medium, No. 108, transystem^®^, HAIN. Lifescience/Copan) taken in a circular motion around the double cone, which is directly anchored to the Endo-Fix-Stem. The swab was inserted deep into the inner lining until resistance was felt. The inner lining consists of soft tissue and is the space around the endoprosthetic parts which are not fixed in the bone (Juhnke et al., 2015; Ranker et al., 2020). A medical doctor took the swabs. Swabs were sent to the microbiology department immediately after sampling. Culture conditions were set according to national guidelines for microbiological diagnostic with an overall incubation time of 14 days. Matrix Assisted Laser Desorption/Ionization Time-of-Flight (MALDI TOF) Mass Spectrometry Analysis or other specific tests were mainly used for identifying bacterial isolates. For this analysis we classified the taxa into two groups, Gram-positive and Gram-negative bacteria. Gram-positive bacteria were distinguished into *Staphylococcus aureus, Staphylococcus* spp*., Streptococcus* spp., *Corynebacteria*, and other Gram-positive bacteria. Gram-negative bacteria were differentiated into *Pseudomonas* spp., *Acinetobacter* spp*., Enterobacteriaceae*, and other Gram-negative bacteria. Finally, we documented the incidence of yeasts.

### Laboratory values

A qualified nurse took blood samples during the inpatient stay and during the visit at our outpatient clinic. After disinfection of the skin, and venous blood stasis, the venous puncture was performed with a 20G needle. The samples were sent to our clinical chemistry department for standard processing. The infection laboratory includes c-reactive protein (CRP; reference is ≤5 mg according to the specifications of our laboratory) in milligram per liter (mg/l), and leucocytes in thousand per microliter (tsd/μl; reference between 3.9 and 10.2 tsd/μl) as well as hemoglobin (Hb) in gram per deciliter [g/dl; reference between 13.5 and 7.2 (g/dl)], and the body temperature in Celsius degree (°C). Body temperatures between 37.5 and ≤38°C are considered elevated body temperature. A body temperature of >38°C is defined as fever. A nurse measured the body temperature with a digital ear thermometer. A daily reporting system protocoled the laboratory values *via* our hospital's internal software. All patients were instructed to clean their stoma from the fifth postoperative day on, one to two times a day, with clear water and optional non-alcoholic soap. Swimming in stationary waters like lakes and swimming pools was not allowed. Swimming in the sea and rivers was allowed. We did not consider these parameters during data collection.

### The classification of infection

We used the international accepted classification of stomal infections by Al Muderis et al. (2016). In this two-center cohort study, the authors divided the level of severity of infection into low and high-grade soft-tissue infection, as well as into bone infection and implant failure. Except implant failure, the groups were separated into subunits A-C; A includes oral antibiotics, B parenteral antibiotics, and C surgical intervention. Implant failure contains parenteral antibiotics and explanation without dividing into subgroups.

### Data collection and loss to follow-up

**Figure 1:**
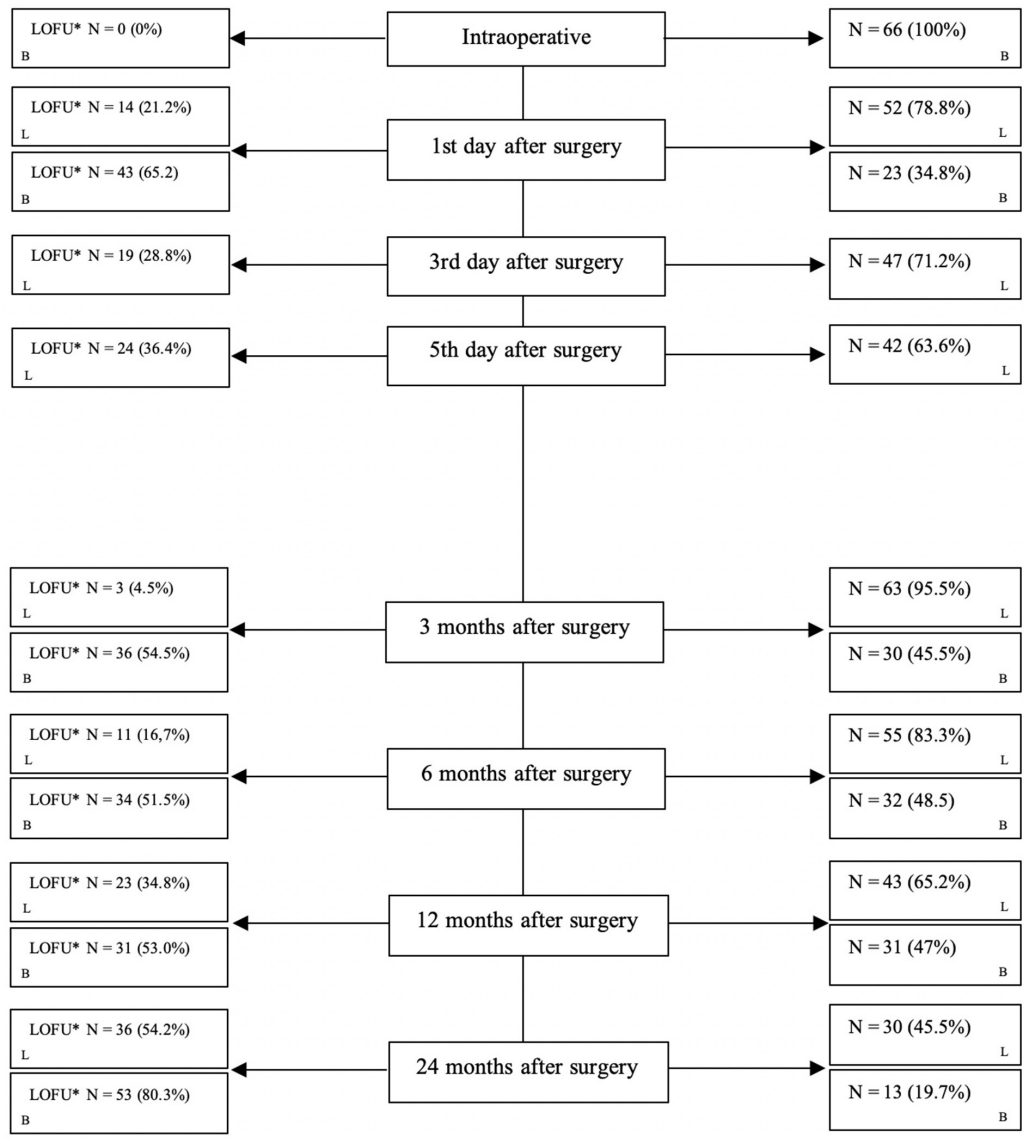
Loss to follow-up during each step. *LOFU, loss to follow-up; N, number; L, laboratory value; B, bacterial swabs.

Within a standardized protocol clinical data from each patient were systematically collected. Included are every result of the standardized stomal swabs and infection laboratory values. Loss to follow-up is shown in Figure 1.

### Statistical analysis

In August 2021, all relevant data of the study hypothesis were systematically evaluated. Descriptive analysis was performed for the stomal swabs, and the infection laboratory values. Besides, the descriptive evaluation of bacterial colonization on the stoma, all infections were numerically captured and underlying the causing bacterial taxa. The whole dataset was checked for normal distribution. Statistical analysis was performed using SPSS 28 (IBM, SPSS Inc., Chicago, IL). Subgroup analysis was carried out according to Gram-staining of bacteria and follow-up of patients.

## Results

### Main results

From 2017 to 2019, *n* = 66 patients underwent the TOPS procedure after transfemoral amputation in our tertiary care hospital in northern Germany (Örgel et al., 2022). Over time, the loss to follow-up increased as expected. Figure 1 shows in detail the loss to follow-up over 24 months.

The mean age of the patients was 50.8 ± 12.3 years (Örgel et al., 2022). One Patient died due to secondary disease. The most common reason for amputation was trauma. With regard to the cohort up to the 13 months follow-up, 7.9% (five patients) developed infections surrounding the TOPS procedure. In relation to the whole cohort with loss to follow-up, of 80.3% at the 24 months follow-up the infection rates increased up to 38.3%. Detailed information about the cohort is shown in Table 1.

**Table 1:** Demographic data of the whole cohort.

	**TOPS cohort (*n* = 66)**
**Sex no. (%) (Örgel et al.**, **2022****)**	
Male	37 (56.1)
Female	29 (43.9)
**Side no. (%)**	
Left	34 (51.6)
Right	28 (42.4)
Both side	4 (6.1)
**Reason for amputation no. (%) (Örgel et al.**, **2022****)**	
Trauma	46 (69.7)
Tumor	5 (7.6)
Vascular disease	6 (9.1)
Sepsis	1 (1.5)
Iatrogenic^*^	8 (12.1)
Age (years) mean ± SD (95%-CI) (Örgel et al., 2022)	50.8 ± 12.3 (47.8–53.9)
BMI (kg/m^2^) mean ± SD (95%-CI) (Örgel et al., 2022)	26.9 ± 6.3 (25.4–28.5)
**Secondary diseases no. (%)**	
Arterial hypertension	5 (7.6)
Peripheral arterial occlusive disease	3 (1.5)
Depression	2 (2.9)
Chronic obstructive pulmonary disease	2 (2.9)
Diabetes mellitus	2 (2.9)
Coronary heart disease	2 (2.9)
Sudeck's disease	1 (1.5)
Rheumatoid arthritis	1 (1.5)
Atrial fibrillation	1 (1.5)

* Iatrogenic: secondary amputation because of complications after elective surgery like periprosthetic knee-joint infection or failed osteosynthesis.

**Figure 2:**
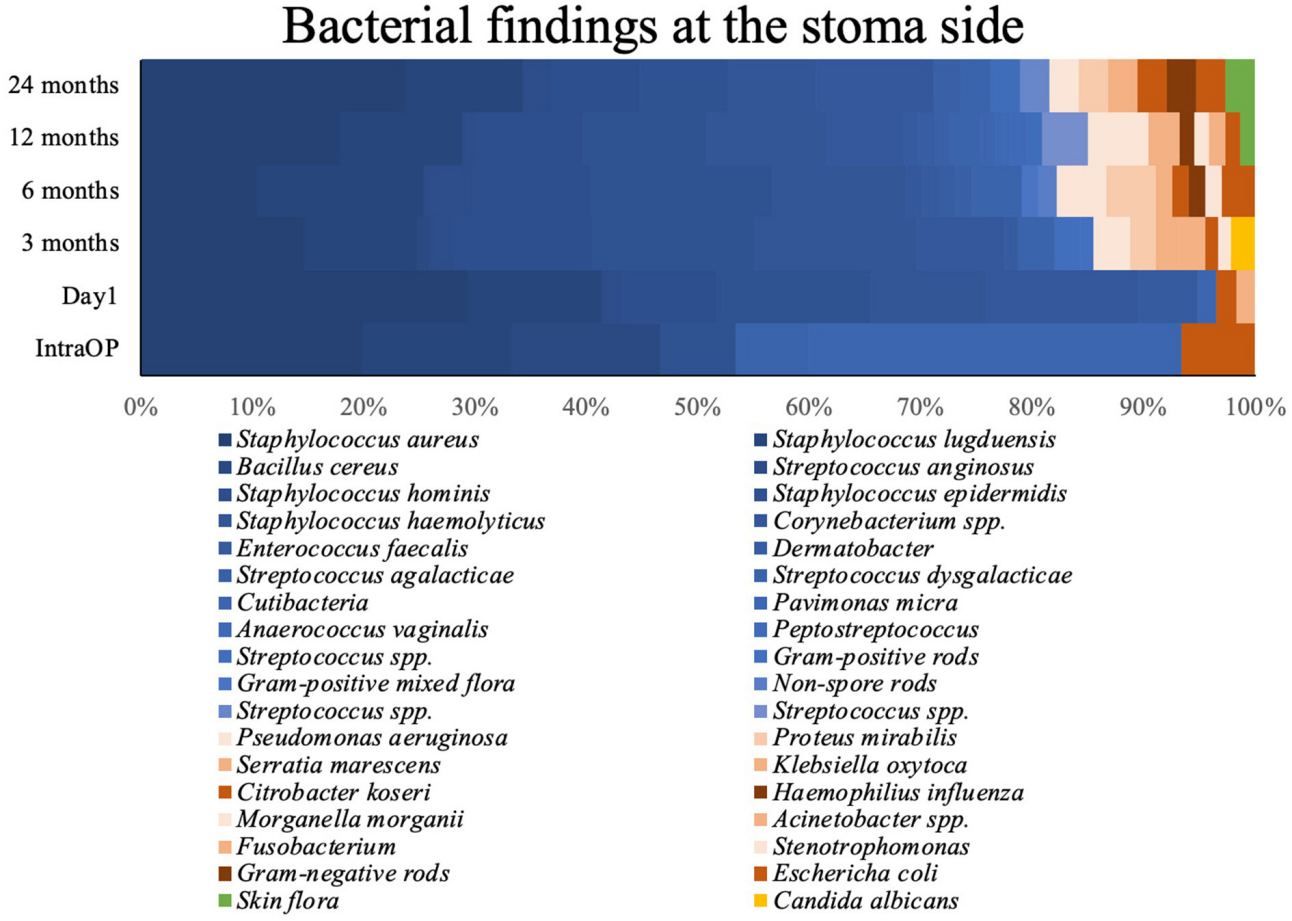
Bacterial findings at the stomal side during the observed period. Blue, GPB; red, GNB; yellow, Candida albicans; green, Skin flora.

Figure 2 shows the results of the microbiological examination of the swabs. During every sampling of the 24 months follow-up the most common taxa found at the skin implant interface were *Staphylococcus aureus, Staphylococcus* spp., and *Streptococcus* spp. (Örgel et al., 2022). None of the *Staphylococcus aureus* were Oxacillin or Methicillin-resistant. After the first day, we noted a rare count of Gram-negative bacteria with a fairly stable proportion over the entire study period. At the 3 months follow-up, we had two swabs identifying *Candida albicans*.

Detailed information is shown in Figure 2 and Table 2. Figure 3 shows the long-term course of the infection values [CRP (mg/l), leucocyte (tsd/μl)], hemoglobin (g/dl), and body temperature (°C).

**Table 2:** Presentation of the most common bacterial species at the intervals “during the surgery (IntraOP)”, “first day after surgery (Day1)”, “3, 6, 12, and 24 months (m) after surgery”.

**Bacteria**	IntraOP^*^	Day1^*^	3M^*^	6M^*^	12M^*^	24M^*^	Total^*^
**Gram-positive bacteria (GPB) no. (%)**							
*Staphylococcus aureus*	3 (20.0)	17 (29.3)	13 (14.9)	7 (10.4)	13 (18.1)	9 (24.3)	62 (21.1)
*Staphylococcus* spp.*^#^*	3 (20.0)	26 (44.8)	46 (52.9)	36 (53.7)	31 (43.1)	13 (35.1)	155 (52.7)
*Streptococcus* spp.*^*##*^*	1 (6.7)	1 (1.7)	7 (8.0)	7 (10.4)	8 (11.1)	5 (13.5)	29 (9.9)
*Corynebacterium* spp.	0 (0.0)	8 (13.8)	7 (8.0)	1 (1.5)	5 (6.9)	4 (10.8)	25 (8.5)
Other Gram-positive species^###^	7 (46.7)	4 (6.9)	3 (3.4)	4 (6.0)	5 (6.9)	0 (0.0)	23 (7.8)
Total GPB (Örgel et al., 2022)	14 (93.3)	56 (96.6)	76 (87.4)	55 (82.1)	62 (86.1)	31 (83.8)	294 (87.5)
**Gram-negative bacteria (GNB) no. (%)**							
*Pseudomonas* spp.	0 (0.0)	0 (0.0)	3 (3.4)	3 (4.5)	4 (5.6)	1 (2.7)	11 (26.2)
*Acinetobacter* spp.	0 (0.0)	1 (1.8)	0 (0.0)	0 (0.0)	0 (0.0)	0 (0.0)	1 (2.4)
*Enterobacteriaceae* spp.^*####*^	1 (6.7)	1 (1.8)	3 (3.4)	4 (6.0)	1 (1.4)	3 (8.1)	13 (31.0)
Other Gram-negative species^######^	0 (0.0)	0 (0.0)	5 (5.7)	5 (7.5)	5 (6.9)	2 (5.4)	17 (40.5)
Total GNB (Örgel et al., 2022)	1 (6.7)	2 (3.4)	11 (12.6)	12 (17.9)	10 (13.9)	6 (16.2)	42 (12.5)
Total bacteria No. (%)	15 (^*^100/^**^4.5)	58 (^*^100/^**^17.3)	87 (^*^100/^**^25.9)	67 (^*^100/^**^19.9)	72 (^*^100/^**^21.4)	37 (^*^100(^**^11.0)	336 (^**^100)
Total swabs No. (%)	66 (^***^33.8)	23 (^***^11.8)	30 (^***^15.4)	32 (^***^16.4)	31 (^***^16.9)	13 (^***^6.7)	195 (^***^100)

* Denotes 100% of the bacteria of each follow-up.

** Denotes the percentage proportion of the total number of bacteria.

*** Denotes the percentage proportion of the total number of bacteria.

# Includes: *Staphylococcus lugdunensis, Staphylococcus epidermidis, Staphylococcus haemolyticus, Staphylococcus hominis*.

## Includes: *Streptococcus anginosus, Streptococcus agalactiae, Streptococcus dysgalactiae*, Non-hemolytic Streptococcus, Hemolytic Streptococcus, Pepto streptococcus.

### Includes: *Bacillus cereus, Enterococcus faecalis, Cutibacteria, Dermabacter, Parvimonas micra, Anaerococcus vaginalis*, Gram-positive rods, mixed flora with Gram-positive bacteria, non-spore rods.

#### Includes: *Eschericha coli, Klebsiella oxytoca, Citrobacter koseri*.

##### Includes: *Proteus mirabilis, Serratia marcescens, Morganella morganii, Fusobacterium, Stenotrophomonas, Haemophilus influenza*, Gram-negative rods.

###### Denotes the variable.

**Figure 3:**
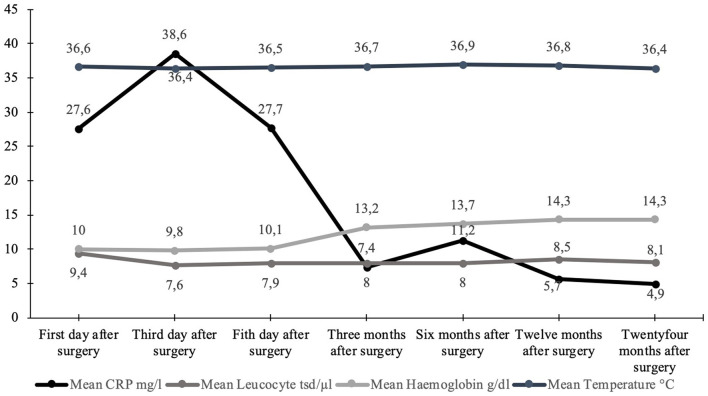
Graphical visualization of the mean values of the long-term course of body temperature (°C), hemoglobin (g/dl), and infection values [leucocytes (tsd/μl) + CRP (mg/l)].

### Infection

Five documented infections occurred. The infections were classified according to Al Muderis et al. (2016). We had one high-grade (2C), two low-grade (1A), one bone infection before the first surgery, and one infection of a hematoma after the first surgery. The infections were cured with species-specific resistogram antibiotic treatment, and in some cases, with additional surgery (abscess splitting). No implant loosening or sepsis was observed. Detailed information is shown in Tables 3, 4.

**Table 3:** Etiology and outcomes of patients with infection.

**Age (years)**	**Time**	Degree of infection^**^	**Localization**	**Micro- biology**	**C-reactive protein mg/L**	**Leucocytes tsd/μL**	**Treatment**
35	2 months after surgery	High grade (2C)^*^, Abscess	The soft tissue near the stoma	*Staphylococcus aureus et Streptococcus pyogenes*	62.5	16.3	Parenteral antibiotics^***^ (Ampicillin/Sulbactam) 10 days, abscess splitting
57	3 months after surgery	Low grade (1A)^*^	The soft tissue surrounding the stoma	*Staphylococcus aureus*	32.0	6.3	Oral antibiotics^***^ (Ampicillin/Sulbactam) 10 days
50	3 months after surgery	Low grade (1A)^*^	The soft tissue surrounding the stoma	Coagulase negative staphylococci, Gram positive coryneform rod, *Serratia liquefaciens*	31.9	10.4	Per os and parenteral antibiotics^***^ (Clindamycin + Piperacillin + beta-lactam) 10 days
32^**^	Before the first surgery^##^	Bone infection (3)^*^	intramedullary	*Propionibacterium acnes, E. coli*	179.1	8.9	10 days Parenteral antibiotics^***^ (Meropenem), surgery
39^**^	3 days after the first surgery^#^	Infection of hematoma	soft tissue	*Staphylococcus aureus*	235.9	15.2	10 days Parenteral antibiotics^***^ (Cephalosporin), surgery

* According to Al Muderis et al. (2016).

** The patient had many surgeries before in their home country (Middle East) due to amputation by a shot fracture.

*** Antibiotics were given according to species-specific resistogram.

# Two-step procedure—no stoma was created until the infection occurred.

## This means that several previous surgeries were necessary before 1st step surgery could be performed.

**Table 4:** Number of complications.

	**TOPS cohort with complications (*n* = 6)**
Death no. (%)	1 (1.5)
Infection no. (%)^*^	
Total	5 (7.6)
Low grade tissue infection	2 (2.9)
High grade tissue infection	1 (1.5)
Bone infection	1 (1.5)^**^
Hematoma infection	1 (1.5)^***^

* Infection after 2nd surgery of TOPS procedure;

** The infection occurs before the first surgery;

*** The infection occurs after the first and before the second surgery.

## Discussion

One of the most frequent questions in the context of TOPS concerns the risk of infections due to the lack of a physiological skin barrier of the stoma. Skin flora has several functions. This circumstance includes the protective function against pathogen invasion, the development and establishment of the immune system, and the catabolism of natural products (Kong and Segre, 2012; Scharschmidt and Fischbach, 2013; Belkaid and Segre, 2014). In addition to fungi, viruses, archaea, and small arthropods, the normal skin flora consists of millions of bacteria (Byrd et al., 2018; Swaney and Kalan, 2021). This prospective data analysis showed infection rates of 7.9% at the 3 months follow-up. With regard to our 24 months follow-up, the infection rate increases up to 38.5% according to the loss to follow-up of 80.3%. This circumstance is concordant to the literature, which reports infection rates ranging from 5.1 to 68.2% and the null hypothesis could be accepted.

The stoma of TOPS is mainly colonized by microbiological organisms of the normal skin flora (Cundell, 2018). *Staphylococcus aureus, Staphylococcus* spp., and *Streptococcus* spp. are the most common identified bacterial species. Subsequently, the findings were split into Gram-positive and Gram-negative bacteria. Gram-positive was the most common taxa at the stoma. This fact confirms existing results. Beck et al. showed comparable findings in their prospective longitudinal scientific work without infection prevalence. They made a slightly different classification than we did. In their study, staphylococci, streptococci, and corynebacteria were the dominant taxa (Beck et al., 2019).

Regarding the taxa of the normal skin flora, we did not identify Propioniebacteria in the stomal area, which also belong to the main taxa of normal skin flora. Cutibacterium is more likely to be found in dry and sebaceous regions than in the region of the thigh skin (Byrd et al., 2018; Timm et al., 2020). Contrary to our protocol, Beck et al. acquired a microbiological swab from the skin surface of the ipsilateral and the opposite side and a stomal swab.

The distribution of bacterial species between the contralateral and ipsilateral thighs was comparable (Beck et al., 2019). Oh et al. published results in which they analyzed the structural and functional constitution of the human skin microbiome prospectively and longitudinally. They took swabs from various body sites, such as the glabella, external auditory canal, manubrium, antecubital fossa, or inguinal crease, popliteal fossa, the toe box, and plantar fossa (Schommer and Gallo, 2013; Oh et al., 2016; Cundell, 2018; Timm et al., 2020). In addition to virus and eukaryota, they equally identify bacterial taxa as shown by Beck et al. and the present results (Oh et al., 2016; Beck et al., 2019). With increasing time after creating the stoma, an equilibrium of the bacterial flora with the leading taxa Staphylococci developed, which could have a protective effect. In the course of 24 months we could not detect any specific bacterial pathogen. Our patients with TOPS had no attributable risk during the observed period with a probable biofilm formation of *Staphylococcae*, or yeasts. *Enterobacterales* were only rarely detected and in equal counts over the 3–24 months screening. *Pseudomonadaceae*, as a potential pathogenic species for chronic wound infections and often linked with a humid environment, were scarcely found in our microbiological cultures of the swabs without any attributable infection. Concerning dental implants, which are the origin of the development of TOPS, Lafaurie et al. showed in their systematic review that the taxa differ in peri-implantitis, periodontitis, and healthy implants.

Therefore, it is hypothesized that stable bacterial flora at the stoma side, according to the normal skin flora, could inhibit infections, so regular stoma hygiene (without a specific disinfection protocol) is required to obtain a stable bacterial flora. This circumstance is consistent with the results of our study, as we had only three minor infections after creating the stoma, which could be cured with antibiotics. The other two infections (Table 3) had a presumptive predictive increased risk of infections because the patients had multiple pre-surgeries in the Middle East after sustaining war injuries (Murray et al., 2009, 2011a,b; Al Muderis et al., 2016), although the laboratory values showed a normal dynamic for each (Figure 3). Our study concludes that a stable bacterial flora might have a protective function regarding pathogens with high infection risk. Although patients treated with TOPS will have a permanent stoma, it seems that during maturation and regaining a stable balance of different physiological taxa, the risk for deep infections is much lower than expected.

### Limitations

This study shows the results of a 24-month follow-up period, but it is limited in showing long-term infectious complications and does not allow a differentiation between short- and long-term complications, and possible varying pathogens. No control group was included.

Moreover, the infection rates are calculated for the whole cohort. Loss to follow-up was 65.2% on the first day after surgery and 80.3% after 24 months after surgery. This aspect was considered when interpreting the results. In addition, this circumstance is caused by the fact that some patients visited our follow-up appointments irregularly due to minor compliance. However, this study is the first prospective study including TOPS patients, screening their bacterial taxa at the stoma side over 24 months.

## Conclusion

Despite the permanently open environment of the stoma with its bacterial taxa and the consecutive connection to the prosthesis, the stoma of our cohort shows a dominated stable Gram-positive taxa with an infection rate of 7.9% at the 3 months follow-up and 38.5% at the 24 months follow up. Thus, this study shows that the soft tissue inside and around the transcutaneous stoma is immediately after surgery colonialized by multiple taxa which are changing over time. The stable Gram-positive dominated bacterial taxa could be seen as a protective factor against ascending periprosthetic infections and could possibly explain the relatively low infection rate in this study as well as in literature.

## Data availability statement

The raw data supporting the conclusions of this article will be made available by the authors, without undue reservation.

## Ethics statement

All procedures performed in studies involving human participants were in accordance with the ethical standards of the institutional and/or national research committee and with the 1964 Helsinki Declaration and its later amendments or comparable ethical standards. Due to the retrospective data collection ethical approval was given as a waiver (No. 10513_BO_K_2022) and consent was granted by the Ethics Committee of Hannover Medical School.

## Author contributions

Conceptualization: MÖ and AR. Data curation: MÖ, SR, H-HA, AR, and TG. Formal analysis: MÖ, LS, and H-HA. Funding acquisition. Investigation: MÖ, SR, AR, and TG. Methodology: MÖ, LS, CK, SR, and AR. Project administration: MÖ, SR, and LS. Resources and writing—original draft: MÖ and SR. Software: MÖ, LS, CK, and AR. Supervision: MÖ, LS, and AR. Validation: MÖ, SR, LS, AR, and TG. Visualization: MÖ, AR, and TG. Writing—review and editing: MÖ, LS, CK, H-HA, and AR. All authors contributed to the article and approved the submitted version.

## Conflict of interest

The authors declare that the research was conducted in the absence of any commercial or financial relationships that could be construed as a potential conflict of interest.

## Publisher's note

All claims expressed in this article are solely those of the authors and do not necessarily represent those of their affiliated organizations, or those of the publisher, the editors and the reviewers. Any product that may be evaluated in this article, or claim that may be made by its manufacturer, is not guaranteed or endorsed by the publisher.
